# Supplementary material for: Predicting Intraoperative Hypothermia Burden during Non-Cardiac Surgery: A Retrospective Study Comparing Regression to Six Machine Learning Algorithms
Source: J Clin Med. 2023 Jun 30;12(13):4434. doi: 10.3390/jcm12134434 (PMC10342641; doi:10.3390/jcm12134434)

Predicting intraoperative hypothermia burden during non-cardiac surgery: a retrospective study comparing regression to six machine learning algorithms

Supplemental Tables

Supplemental Table S1: Initial parameters and hyperparameter search space of the applied machine learning algorithms.

<table>
<tr><td colspan="3">The definition are given as JSON objects, as per `scikit-optimize` specification (https://scikit-optimize.github.io/stable/auto_examples/sklearn-gridsearchcv-replacement.html). Precise definitions of all parameters, and their possible effect on the prediction model can be found in the documentation of each prediction algorithm on `scikit-learn` web page, e.g.,
https://scikit-learn.org/stable/modules/generated/sklearn.neural_network.MLPClassifier.html</td></tr>
<tr><th>Prediction algorithm</th><th>Initial parameters</th><th>Search space</th></tr>
<tr><td>Logistic regression</td><td>{
'solver': 'saga',
'class_weight': 'balanced',
}</td><td>{
'clf__C': (1e-6, 100.0, 'log-uniform'),
'clf__max_iter': [100, 200, 500, 1000],
'clf__penalty': ['l1', 'l2', 'elasticnet'],
'clf__l1_ratio': (0, 1),
}</td></tr>
<tr><td>Gaussian Naïve Bayes</td><td>default</td><td>{
'clf__var_smoothing': (1e-10, 1e-7)
}</td></tr>
<tr><td>Linear Discriminant Analysis</td><td>default</td><td>{
'clf__solver': ['svd', 'lsqr'],
}</td></tr>
<tr><td>K-Nearest Neighbors</td><td>default</td><td>{
'clf__n_neighbors': (1, 8),
'clf__weights': ['uniform', 'distance'],
'clf__p': [1, 2, 3],
}</td></tr>
<tr><td>Multilayer Perceptron (Feed-forward Neural Network)</td><td>default</td><td>{</td></tr>
</table>

		'clf__hidden_layer_sizes': [(100, 50), (100, 100, 50), (100, 100, 100, 50)],<br>'clf__activation': ['logistic', 'tanh', 'relu'],<br>'clf__learning_rate': ['constant', 'invscaling', 'adaptive'],<br>}
Random Forest (RF)	default	{<br>'clf__max_depth': [10, 20, 40, 60, 80, 100],<br>'clf__max_features': ['auto', 'sqrt', 'log2'],<br>'clf__min_samples_leaf': (1, 5),<br>'clf__min_samples_split': (2, 11),<br>'clf__n_estimators': [20, 50, 100, 200, 400],<br>}
Extremely Gradient Boosting machine (XGBoost)	default	{<br>'clf__learning_rate': (0.01, 1.0, 'log-uniform'),<br>'clf__min_child_weight': (1, 10),<br>'clf__max_depth': (1, 10),<br>'clf__max_delta_step': (1, 20),<br>'clf__subsample': (0.01, 1.0, 'uniform'),<br>'clf__colsample_bytree': (0.01, 1.0, 'uniform'),<br>'clf__colsample_bylevel': (0.01, 1.0, 'uniform'),<br>'clf__reg_lambda': (1, 1000, 'log-uniform'),<br>'clf__reg_alpha': (0.1, 1.0, 'log-uniform'),<br>'clf__gamma': (0.1, 0.5, 'log-uniform'),<br>'clf__n_estimators': [20, 50, 100, 200, 400],<br>}

Supplemental Table S2: Detailed comparison of model discrimination metrics for prediction of hypothermia burden.

	**No Hypothermia**				**Mild Hypothermia**				**Moderate Hypothermia**				**Severe Hypothermia**			
	**AUROC**	**F1**	**Precision**	**Recall**	**AUROC**	**F1**	**Precision**	**Recall**	**AUROC**	**F1**	**Precision**	**Recall**	**AUROC**	**F1**	**Precision**	**Recall**
**XGBoost**	0.781	0.488	0.494	0.483	0.655	0.366	0.366	0.366	0.617	0.325	0.353	0.301	0.812	0.585	0.544	0.633
**Random Forest**	0.756	0.455	0.472	0.439	0.641	0.363	0.358	0.369	0.604	0.300	0.337	0.271	0.784	0.556	0.505	0.619
**MLP**	0.738	0.444	0.450	0.439	0.607	0.345	0.325	0.367	0.582	0.289	0.323	0.262	0.761	0.525	0.504	0.547
**LDA**	0.735	0.423	0.466	0.387	0.626	0.365	0.349	0.383	0.592	0.312	0.332	0.294	0.748	0.525	0.487	0.570
**Logistic Regression**	0.735	0.465	0.428	0.508	0.627	0.329	0.349	0.311	0.594	0.272	0.334	0.230	0.749	0.530	0.477	0.597
**KNN**	0.676	0.382	0.400	0.366	0.568	0.322	0.308	0.339	0.542	0.300	0.295	0.306	0.699	0.443	0.459	0.429
**GNB**	0.673	0.406	0.374	0.443	0.580	0.387	0.292	0.575	0.558	0.138	0.308	0.089	0.694	0.361	0.474	0.292

XGBoost: extreme gradient boosting, MLP: multi-layer perceptron neural network, LDA: linear discriminant analysis, KNN: k-nearest neighbor, Gaussian NB: gaussian naïve bayes

Supplemental Table S3: Comparison of model discrimination metrics for prediction of hypothermia at a single time point.

	< 35 °C				< 35.5 °C				< 36 °C			
	**AUROC**	**F1**	**Precision**	**Recall**	**AUROC**	**F1**	**Precision**	**Recall**	**AUROC**	**F1**	**Precision**	**Recall**
**XGBoost**	0.715	0.013	0.524	0.007	0.717	0.162	0.574	0.094	0.736	0.679	0.669	0.689
**Random Forest**	0.703	0.001	1.000	0.001	0.695	0.005	0.867	0.003	0.719	0.665	0.655	0.675
**MLP**	0.599	0.105	0.164	0.078	0.653	0.258	0.368	0.199	0.676	0.629	0.624	0.634
**LDA**	0.699	0.060	0.325	0.033	0.691	0.084	0.503	0.046	0.702	0.653	0.641	0.665
**Logistic Regression**	0.701	0.187	0.110	0.638	0.692	0.410	0.299	0.650	0.703	0.653	0.641	0.666
**KNN**	0.601	0.028	0.300	0.014	0.622	0.160	0.374	0.102	0.653	0.600	0.610	0.591
**GNB**	0.641	0.173	0.138	0.232	0.645	0.271	0.324	0.233	0.656	0.515	0.663	0.421

XGBoost: extreme gradient boosting, MLP: multi-layer perceptron neural network, LDA: linear discriminant analysis, KNN: k-nearest neighbor, GNB: gaussian naïve bayes

Supplemental Table S4: Comparison of model calibration for prediction of hypothermia at a single time point.

	< 35 °C			< 35.5 °C			< 36 °C		
	**Mean**	**Intercept**	**Slope**	**Mean**	**Intercept**	**Slope**	**Mean**	**Intercept**	**Slope**
**XGBoost**	-0.011	0.103	1.047	0.002	-0.022	0.980	-0.006	-0.007	0.997
**Random Forest**	-0.014	1.159	1.465	-0.004	0.724	1.557	-0.007	-0.009	1.353
**MLP**	-0.036	-2.063	0.151	-0.001	-0.841	0.319	-0.022	-0.022	0.447
**LDA**	-0.048	-0.542	0.798	-0.014	-0.027	0.990	-0.019	-0.022	1.017
**Logistic Regression**	-2.489	-2.697	1.008	-1.276	-1.408	0.972	-0.015	-0.017	0.994
**KNN**	0.135	-2.106	0.079	0.092	-1.114	0.085	0.036	0.004	0.182
**GNB**	-0.797	-2.470	0.063	0.082	-1.258	0.055	0.624	0.099	0.063

XGBoost: extreme gradient boosting, MLP: multi-layer perceptron neural network, LDA: linear discriminant analysis, KNN: k-nearest neighbor, GNB: gaussian naïve bayes

Supplemental Figures

Supplemental Figure S1: Receiver operating characteristic curve for prediction of hypothermia at a single time point. XGBoost: extreme gradient boosting, MLP: multi-layer perceptron neural network, LDA: linear discriminant analysis, KNN: k-nearest neighbor, GNB: gaussian naïve bayes

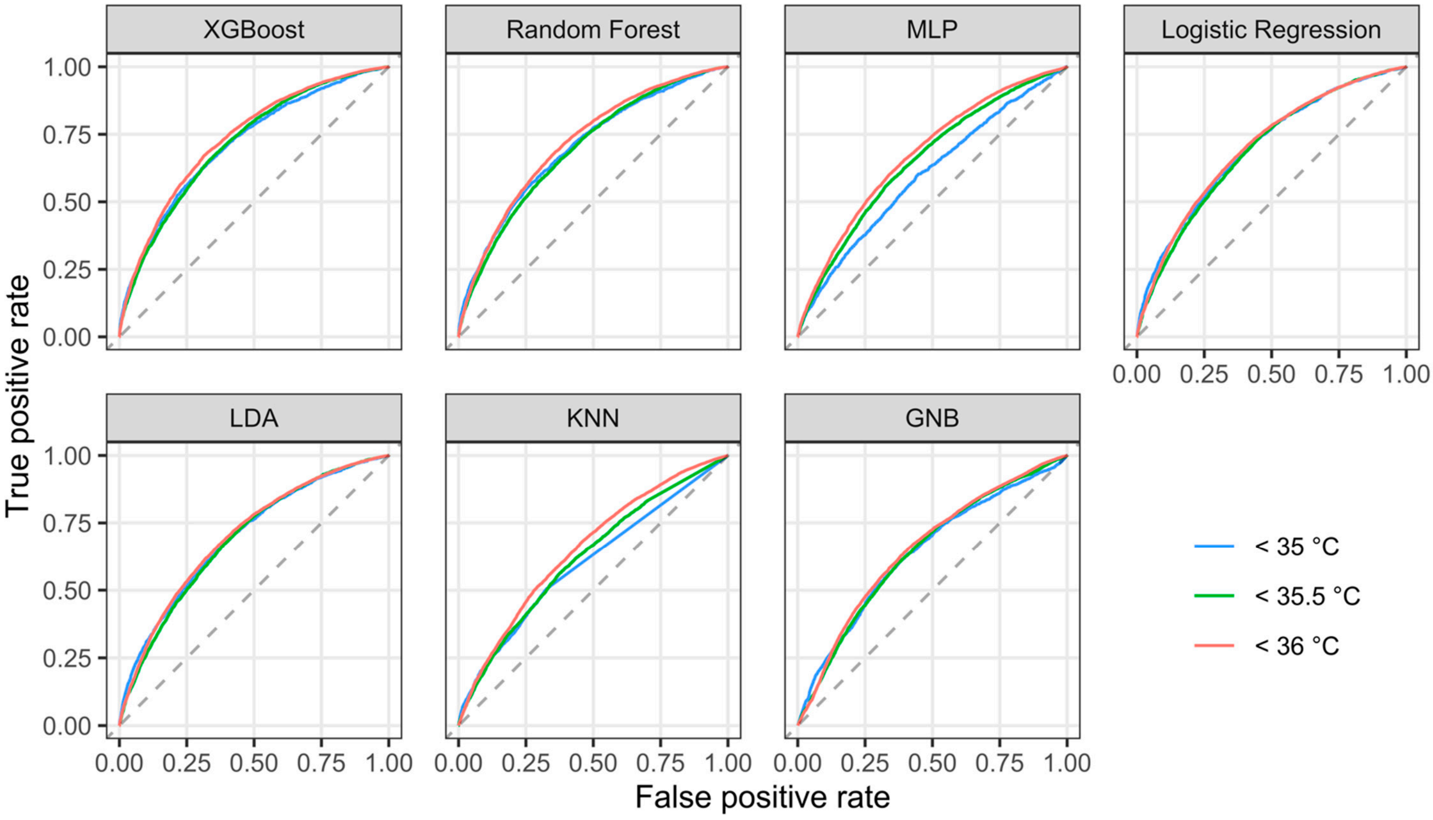

Supplemental Figure S2: Calibration plots for prediction of hypothermia at a single time point. XGBoost: extreme gradient boosting, MLP: multi-layer perceptron neural network, LDA: linear discriminant analysis, KNN: k-nearest neighbor, GNB: gaussian naïve bayes

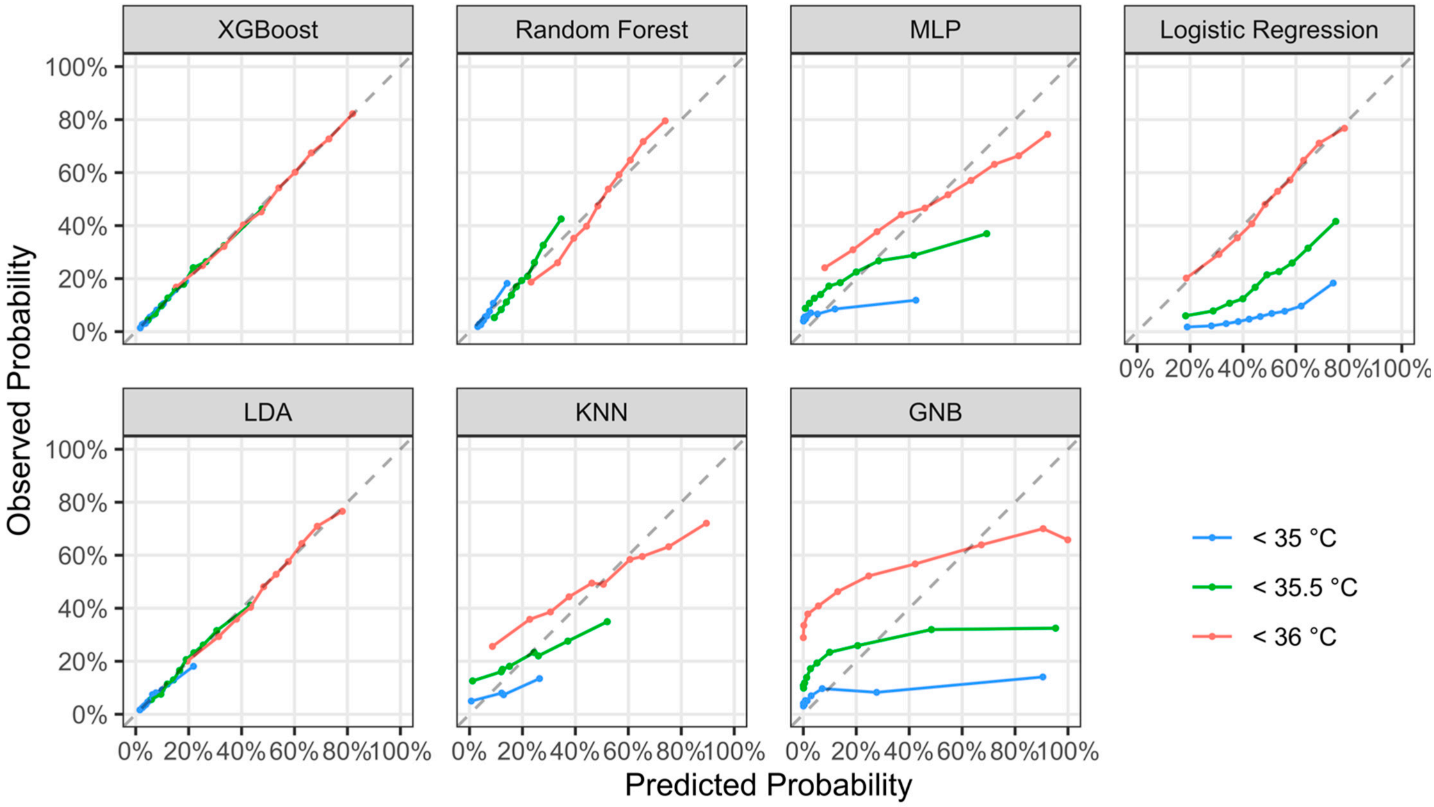

Supplement: Supplementary file 1 [file jcm-12-04434-s001.zip › jcm-2426248-supplementary.pdf]
